# Measles Outbreaks in the Eastern Mediterranean Region: Urgent Need for Strengthened Vaccination Efforts

**DOI:** 10.1007/s44197-024-00227-3

**Published:** 2024-04-25

**Authors:** Jaffar A. Al-Tawfiq, Ziad A. Memish

**Affiliations:** 1 Infectious Disease Unit, Specialty Internal Medicine, Johns Hopkins Aramco Healthcare, Dhahran, Saudi Arabia; 2 Division of Infectious Diseases, Indiana University School of Medicine, Indianapolis, IN USA; 3 Division of Infectious Diseases, Johns Hopkins University, Baltimore, MD USA; 4 King Saud Medical City, Ministry of Health & College of Medicine, Alfaisal University, Riyadh, Saudi Arabia; 5 Hubert Department of Global Health, Rollins School of Public Health, Emory University, Atlanta, GA USA; 6 Kyung Hee University, Seoul, South Korea

Measles is a vaccine preventable illness with a well-known high transmissibility, severe symptoms and potentially high mortality. The Paramyxoviridae family of viruses, which includes the measles virus, is the source of this ancient and highly contagious airborne virus that mostly affects children under five years old. Measles is thought to have one of the highest reproductive numbers (R0) with estimates ranging from 12 to 18 [1]. The high R0 highlights how urgently strong vaccination programs must be put in place in order to achieve herd immunity, which is required to protect individuals who are unable to receive vaccinations for medical reasons.

In low and middle-income countries, measles poses a notable mean case-fatality ratio of 2·2% (95% CI 0·7–4·5) in 1990–2015 [2]. In sub-Saharan Africa and certain parts of Asia, the case fatality rate for measles is approximately 5%, with higher rates observed among refugees. The Global Burden of Diseases, Injuries, and Risk Factors Study 2019 identified four distinct risk factors for measles mortality: underweight, wasting, stunning, and vitamin A deficiency in children [3]. While measles can be prevented through vaccination with measles, mumps, and rubella (MMR), maintaining a vaccination rate of over 95% is crucial to prevent outbreaks by ensuring herd immunity [4]. In 2015, all 22 nations in the World Health Organization’s (WHO) Eastern Mediterranean Region (EMR) made a collective pledge to attain measles elimination by 2020. Despite notable achievements in four countries, the majority within the region have not yet reached the goal of measles elimination. Despite global initiatives such as the WHO Global Vaccine Action Plan, aimed at extending the full benefits of immunization to all individuals irrespective of their origin or circumstances, immunization coverage for numerous vaccines reached a plateau even before the onset of the COVID-19 pandemic. Throughout the COVID-19 pandemic, there has been a significant decline in routine immunization rates across different regions of the globe. Efforts to enhance coverage continue to face significant hurdles [5]. By February 2024 measles outbreaks have been reported in all WHO regions, including the EMR (Fig. 1) [6]. The general public is experiencing "fatigue" from the COVID-19 pandemic, and one of the terrible side effects on health that has resulted from the pandemic is the disruption of childhood immunization programs [7]. The impact of COVID-19 on measles vaccination in the EMR has been significant, with cumulative 12.7 million measles-containing vaccine (MCV) zero-dose children in EMR since the pandemic started. Fifty percent were in Pakistan and Afghanistan and the remaining were mostly in Somalia, Yemen, Iraq, Sudan, and Syria. A region with many of its countries, are fragile and conflict-affected states are facing huge challenges in maintaining adequate vaccination coverage against measles. The complex humanitarian situations, including displacement, limited access to healthcare services, and disrupted supply chains, exacerbate the difficulties in delivering vaccination programs effectively. As a result, immunization rates in these countries may be lower than in more stable regions, leading to increased susceptibility to measles outbreaks.

**Fig. 1:**
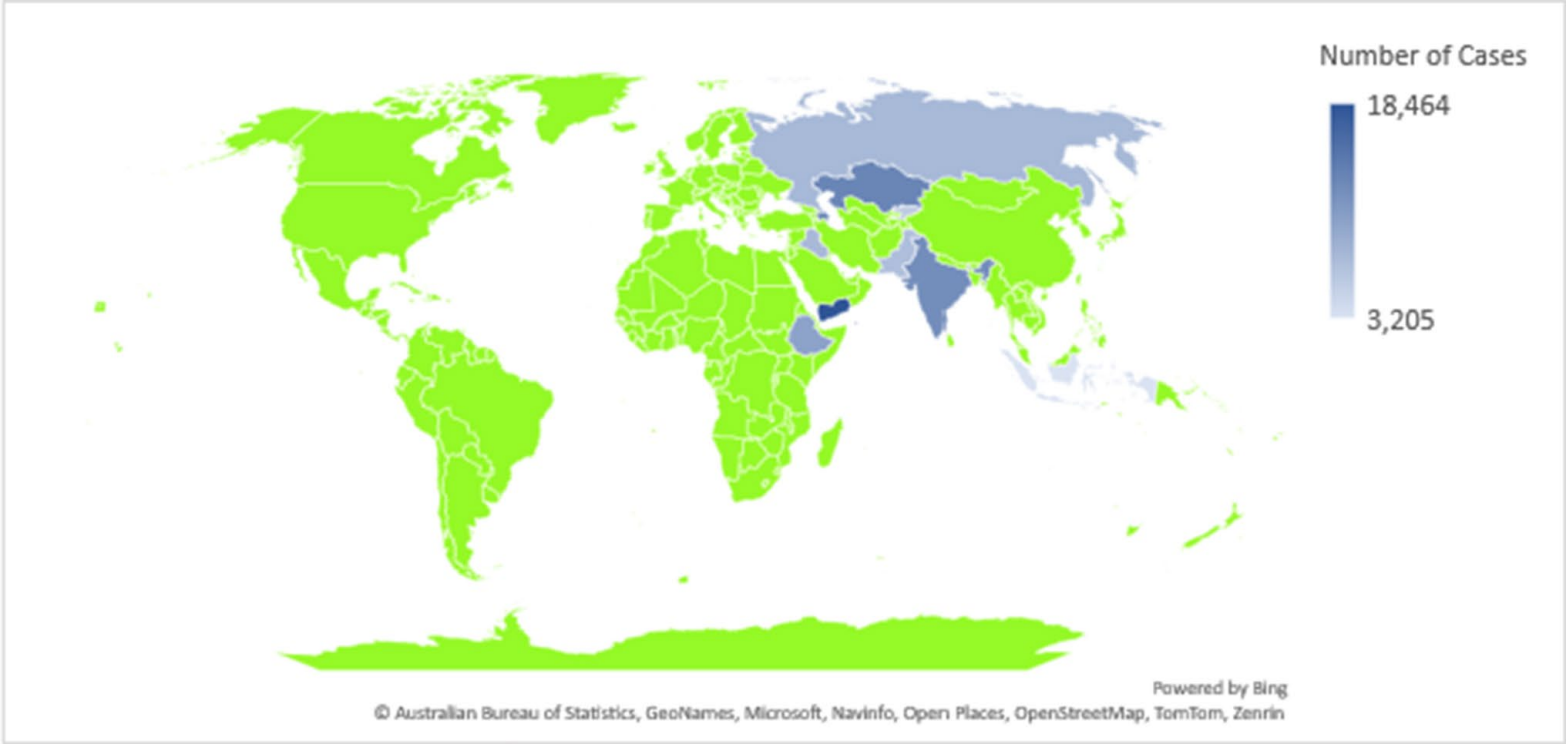
A map of the Eastern Mediterrian Region showing the number of reported Measles Cases as of March 2024 [6]. Data from: https://www.cdc.gov/globalhealth/measles/data/global-measles-outbreaks.html

Between 2019 and 2024, 148,271 cases of measles were reported in EMR. Of which 123,776 confirmed measles cases and 563 deaths were reported in 2023. When all confirmed measles cases are reviewed, 70% of cases are either not or incompletely vaccinated and 15% vaccination status unknown. And the age distribution of cases showed 16% under the age of vaccination, 72% under 5 years and 89% under 10 years of age.

It is critical that steps are taken to prioritize and step-up vaccination campaigns in the impacted nations in order to address this urgent problem. Among the strategies to use are bolstering the infrastructure of healthcare systems. In areas confronting difficulties like conflict or resource scarcity, investing in healthcare systems is essential to enhancing vaccine accessibility and delivery. This entails setting up immunization clinics in underprivileged areas, educating medical staff about immunization procedures, and maintaining a reliable vaccine supply chain [8]. When model trajectories with and without the measles vaccination campaigns in Somalia in 2019 and 2020 are compared, it can be seen that the immunization efforts prevented 72,000 (95% CI 58,000–86,000) case reports between 2019 and 2023 [9].

For an outbreak to be effectively contained, measles cases must be reported promptly and accurately. Enhancing data collection and surveillance systems can aid in tracking the virus’s spread, identifying regions with low vaccination rates, and directing focused interventions. In order to rebuild public confidence in vaccinations, vaccine hesitancy must be addressed [10]. To address misinformation and misconceptions surrounding vaccines, comprehensive community engagement programs, public awareness campaigns, and effective communication strategies should be implemented. This entails using reputable community leaders, religious leaders, and medical professionals as immunization advocates. Enhancing immunization programs entails making sure there is a strong supply chain, sufficient vaccine stockpiles, and a skilled medical staff that can efficiently handle outbreaks and administer vaccinations. It also entails putting in place top-notch monitoring and evaluation systems, regular vaccination campaigns, and catch-up immunization programs for underprivileged populations.

To contain measles outbreaks and stop their further spread, international cooperation and support are essential in addition to the national efforts of the affected countries. International health agencies, like the WHO, ought to keep offering impacted nations technical support, materials, and advice so they can improve their immunization campaigns and contain outbreaks. To guarantee vaccine availability and the execution of comprehensive immunization programs, donor nations and organizations must provide financial support [11]. It is imperative that measles outbreaks are addressed as soon as possible. Inaction runs the risk of undoing the gains made in the fight to eradicate the measles. It is our duty as researchers and healthcare professionals to promote more funding for immunization programs and to give vulnerable populations’ health and wellbeing top priority. Planning for the big catchup measles vaccination campaign is the way forward. There are multiple challenges facing such campaigns including: identifying missed and partially vaccinated children, changing the practice/behavior of healthcare workers and caregiver to vaccinate the older children, recording and reporting coverage data for the new age groups, vaccine availability, high operational costs in certain countries, and finally and most importantly instability and insecurity in some countries.

Despite the regional obstacles, measles is being eliminated in 11 (50%) of the 22 EMR countries, and in four (18%) of those countries, the elimination has been confirmed [12]. These four nations share several notable traits, such as health ministries dedicated to eradicating the measles, consistently high vaccination rates (≥ 95%), robust laboratory support, and surveillance systems. A further seven nations—Kuwait, Morocco, Palestine, Qatar, Saudi Arabia, Tunisia, and United Arab Emirates—have low measles incidence, high rates of measles vaccination, and excellent surveillance, all of which indicate that measles is almost completely eradicated [12].

In conclusion, the recent measles outbreaks in the Middle East and other parts of the globe call for immediate attention and concerted action. Strengthening vaccination efforts, improving healthcare infrastructure, combatting vaccine hesitancy, and fostering international collaboration are crucial steps towards controlling and eliminating measles in this region and beyond.

## Data Availability

Not available as this is an editorial.

## Abbreviations

R0: Reproductive numbers
MMR: Measles, mumps, and rubella
WHO: World Health Organization’s
EMR: Eastern Mediterranean Region
MCV: Measles-containing vaccine
